# A semantic segmentation method to analyze retinal vascular parameters of diabetic nephropathy

**DOI:** 10.3389/fmed.2024.1494659

**Published:** 2024-10-24

**Authors:** Youlv Lu, Ruolin Fang, Bolun Xu, Chunyun Feng, Zhentao Zhu, Meiting Yu, Yuhua Tong

**Affiliations:** ^1^Second Clinical Medical College, Zhejiang Chinese Medical University, Hangzhou, China; ^2^Department of Ophthalmology, The Quzhou Affiliated Hospital of Wenzhou Medical University, Quzhou People’s Hospital, Quzhou, China; ^3^Department of Ophthalmology, Huaian Hospital of Huaian City, Huaian, China

**Keywords:** optical coherence tomography, urinary protein creatinine ratio, full pixel semantic segmentation method, diabetic nephropathy, vascular structure

## Abstract

**Introduction:**

By using spectral domain optical coherence tomography (SD-OCT) to measure retinal blood vessels. The correlation between the changes of retinal vascular structure and the degree of diabetic nephropathy is analyzed with a full-pixel Semantic segmentation method.

**Methods:**

A total of 120 patients with diabetic nephropathy who were treated in the nephrology department of Quzhou People’s Hospital from March 2023 to March 2024 were selected and divided into three groups according to the urinary albumin creatinine ratio (UACR). The groups included simple diabetes group (UACR < 30 mg/g), microalbuminuria group (30 mg/g ≤ UACR <300 mg/g) and macroalbuminuria group (UACR ≥300 mg/g). SD-OCT was used to scan the arteries and veins in the superior temporal area B of the retina. The semantic segmentation method built into the SD-eye software was used to automatically identify the morphology and structure of the vessels and calculate the parameters of arteriovenous vessels. The parameters of arteriovenous vessels are as follows: outer diameter of the retinal artery (RAOD); inner diameter of the retinal artery (RALD); arterial wall thickness (AWT); arterial wall to lumen ratio (AWLR); cross sectional area of arterial wall (AWCSA); retinal vein outer diameter (RVOD); retinal vein inner diameter (RVLD); vein wall thickness (VWT); vein wall to lumen ratio (VWLR); cross sectional area of vein wall (VWCSA). Statistical analysis software was used to compare and analyze the parameters of retinal arteriovenous vessels of the three groups.

**Results:**

The study revealed statistically significant differences in RAOD and RALD among the three groups (*p* < 0.05) with the RAOD and RALD of the macroalbuminuria group and microalbuminuria group being lower than those of the simple diabetes group. Conversely, there were no significant differences in AWT, AWLR and AWCSA among the three groups (*p* > 0.05). Additionally, the differences in RVOD and RVLD among the three groups were found to be statistically significant (*p* < 0.05) with the RVOD and RVLD of the simple diabetes group being lower than those of the microalbuminuria group and macroalbuminuria group. No significant differences were observed in VWT and VWL among the groups. Additionally, RVOD and RVLD were weakly associated with UACR (*R* = 0.247, *p* = 0.007; *R* = 0.210, *p* = 0.021). Full-pixel semantic segmentation method combined with OCT images is a new retinal vascular scanning technology, which can be used as a new method for early diagnosis of diabetic nephropathy. The structural changes of retinal vessels can be used to predict the severity of diabetic nephropathy during the development of diabetic nephropathy.

## 1 Introduction

Diabetic nephropathy (DN) is a severe complication of diabetes (DM) resulting from poor blood sugar control over an extended period. It is characterized by tiny vascular lesions. At the same time, diabetic nephropathy causing renal failure is the important cause leading to the terminal uremia. In early diabetic nephropathy patients, hyperlipidemia and high blood sugar are accompanied by an increased abnormal glomerular filtration rate, proteinuria, serum creatinine, blood urea nitrogen index and progressive changes in renal function. In recent years, the diagnosis of diabetic nephropathy has advanced significantly with various methods such as the serology detection of proinflammatory factors (1–5), inflammatory factors (6), and gene diagnosis (7). However, accurately diagnosing the lesions of diabetic nephropathy remains challenging. Retinal and renal vessels share anatomical, physiological and pathological traits. Diabetes-induced hyperglycemia harms microvessels including those in the retina and kidneys. Retinal vessels which are visible without invasive methods can indicate diabetic microangiopathy severity. Many studies (8–10) show a strong link between retinal microangiopathy and diabetic nephropathy progression. Therefore, by measuring changes in retinal blood vessels, the progression of diabetic nephropathy can be indirectly assessed. This study utilizes the full pixel semantic segmentation method to identify individuals with diabetic nephropathy based on retinal vascular parameters. It also examines the various stages of diabetic nephropathy by analyzing retinal vascular structures, abnormal changes in retinal blood vessel structure and the correlation between the severity of diabetic nephropathy.

## 2 Subjects and methods

### 2.1 Ethical approval

A cohort of 120 patients diagnosed with diabetic nephropathy and admitted to the Department of Nephrology at Quzhou People’s Hospital between March 2023 and March 2024, all of whom were aged 50 years or older, were selected for this study. The research protocol was approved by the Research Ethics Committee of Quzhou People’s Hospital and conducted in compliance with the guidelines outlined in the Helsinki Declaration. Informed written consent was obtained from all participants.

### 2.2 General information and grouping

The inclusion criteria of subjects in this study needed to meet the diagnostic criteria for diabetes nephropathy in the 2021 version of the Chinese Guidelines for the Prevention and Treatment of diabetes Nephropathy, exclude kidney diseases caused by other reasons and meet one of the following conditions: (1) two out of three times over a six-month period, the UACR was found to be at least 30 mg/g; (2) GFR less than 60 mL/min/1.73 m^2^ lasts for more than 3 months. Exclusion criteria: (1) type 1 diabetes, gestational diabetes and special type diabetes; (2) acute complications of diabetes (such as diabetic ketoacidosis); (3) chronic nephritis or kidney combined with other diseases that could lead to proteinuria and hematuria; (4) recent use of hormones, immunosuppressants or diuretics; (5) intraocular pressure over 21 mmHg; (6) eye blinding disease history (such as: optic nerve diseases, senile cataracts, history of closure angle glaucoma, open angle glaucoma, anterior uveitis and retinal vascular disease) and poor vision correction operation difficulties; (7) other causes of proteinuria including acute infection, hypertension and obesity. According to the urinary albumin creatinine ratio (UACR), 120 patients with diabetic nephropathy were divided into diabetic non-nephropathy group (35 subjects), diabetic microalbuminuria group (49 subjects) and diabetic macroalbuminuria group (36 subjects). The UACR of patients in the diabetic non-nephropathy group was less than 30 mg/g and the UACR of patients in the diabetic macroalbuminuria group was greater than or equal to 30 mg/g when the UACR of the patients in diabetic microalbuminuria group was greater than or equal to 30 mg/g and less than 300 mg/g.

### 2.3 Routine inspection items

All participants underwent blood pressure, fasting blood glucose, glycated hemoglobin, triglycerides, cholesterol, liver and kidney function, urine routine, ophthalmic intraocular pressure (IOP) measurement, anterior slit-lamp examination and fundus retinal examination.

### 2.4 OCT image acquisition and parameter measurement of retinal vessels

**Figure 1:**
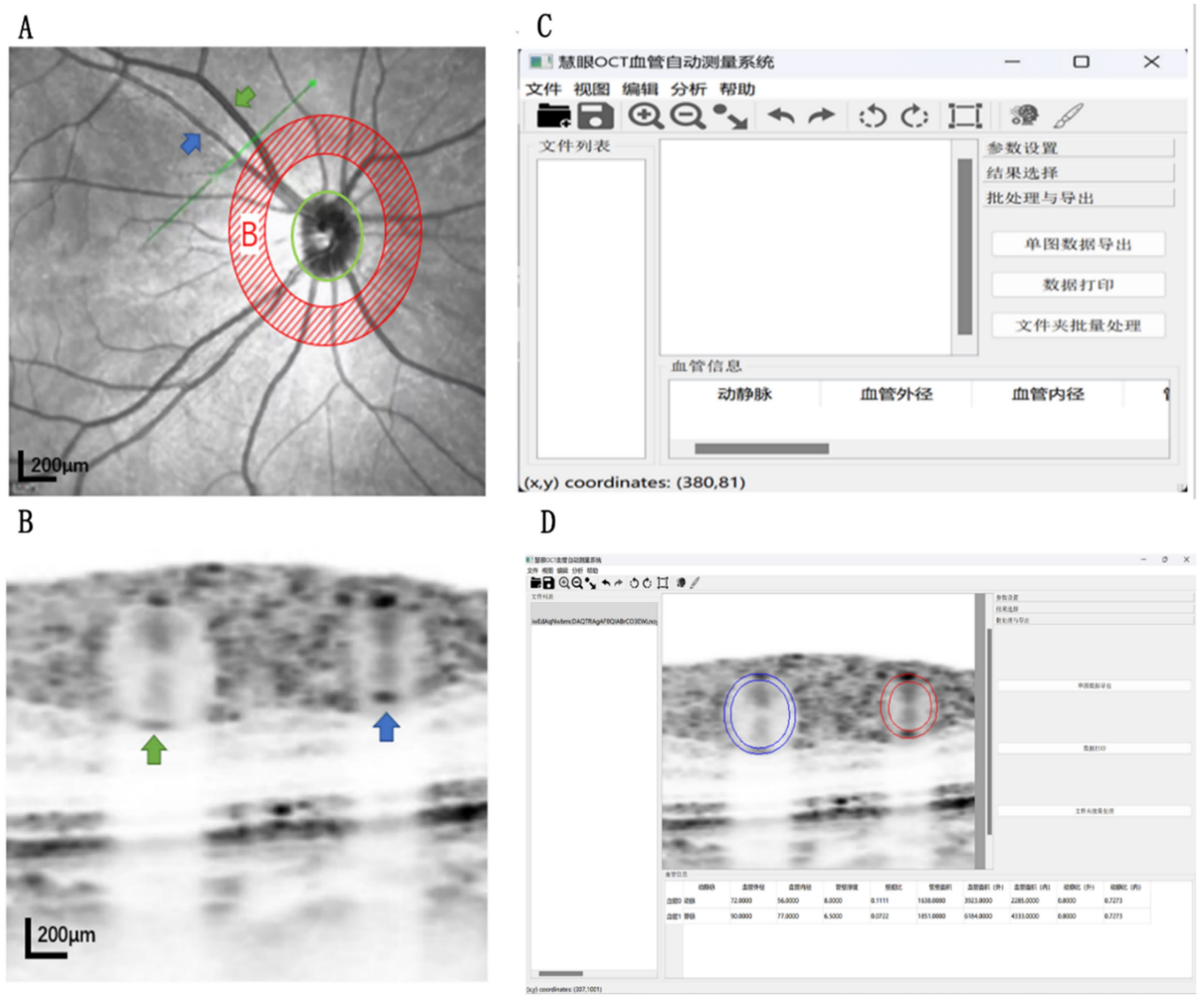
operation process **(A)**: The green straight line represents the OCT linear scan line. the shaded **(B)** area is the linear scan area. When the blue arrow marks the artery, the green arrow marks the vein. B: The image shows the corresponding cross-sectional area image of the arterial and venous vessels in Figure A. **(C)**: The interface is displayed after the initial startup of the Huiyan OCT vascular automatic measurement system **(D)**: the corresponding arterial and venous parameters are measured after importing into the Huiyan software OCT vascular automatic measurement system.

All the study objects of OCT imaging were checked by skilled and highly qualified doctors of ophthalmology through SD-OCT scanning. The subject’s head was fixed on the SD-CT operating table and they were asked to maintain the correct sitting posture during the operation. At the same time, the subject was asked to fixate on the blue fixation light in the lens. The SD-OCT scanner was used to scan the retinal arteries and accompanying retinal veins in the superior temporal region of the right eye of each subject. Zone B was defined as the superotemporal range extending 1–2 disc diameters from the edge of the optic disc. A linear scan with a corresponding depth image was selected for scanning and the scanning line was maintained perpendicular to the vascular axis (Figure 1A). Each eye was scanned three times to select the clearest images of blood vessel walls for the next study. After the scan was completed, the first OCT images of the vertical level ratio was adjusted to 1:1 microns. After 8x magnification, it was saved as a vascular cross-section of a 512 × 512 pixel image (Figure 1B). The image was fed into Eye Recognition Software to identify and measure blood vessels (Figures 1C,D). The software calculated the retinal artery diameter (RAOD) and inner diameter (RALD); vein diameter (RVOD) and inner diameter (RVLD); arterial blood wall thickness (AWT) = (RAOD − RALD)/2; arterial vessel wall to lumen ratio (AWLR) = (RAOD − RALD)/2/RALD; Venous blood wall thickness (VWT) = (RVOD − RVLD)/2; the artery wall cross sectional area (AWCSA) = (RAOD^2^ − RALD^2^) × 3.14/4; wall to lumen ratio of venous vessels (VWLR) = (RVOD − RVLD)/2/RVLD; venous wall cross-sectional area (VWCSA) = (RVOD^2^-RVLD^2^) × 3.14/4.

### 2.5 A new type of image segmentation method

The semantic segmentation method is a method of image processing based on computer vision and deep learning. It is able to classify each pixel in an image into a specific category or region, which enables detailed parsing and recognition of the image content. This method uses a model of training to learn and understand the semantic information in the medical image, specifically the meaning or object representing different areas and applies this understanding to the categorization of each pixel in the image. In the process of semantic segmentation method analysis, each pixel in the image is analyzed according to its features such as color, texture and shape to determine its category and area. In this way, the different objects in the image of the semantic segmentation method can realize accurate positioning and recognition for subsequent image analysis, scene understanding and various applications providing strong support. The application of semantic segmentation method in the recognition and measurement of retinal vascular parameters is a new technology in the field of medical image processing. The recognition software uses the full-pixel semantic segmentation method to identify the image contour of retinal blood vessels. The artery and vein structures in each image can be captured by the software at the same time and the parameters of blood vessels can be automatically calculated and the vascular structural parameters of multiple images can be exported in batches.

### 2.6 Statistical analysis

SPSS 26.0 statistical software was used for data processing and analysis. The measurement data of the three groups exhibited a normal distribution with the mean ± standard deviation utilized to depict the central tendency and variability. Analysis of variance (ANOVA) was employed to compare the groups while frequency (%) was utilized for categorical data. Correlation analysis was conducted to investigate the relationship between urinary albumin creatinine ratio and retinal vascular parameters in patients with diabetic nephropathy when statistical significance was defined as *p* < 0.05.

## 3 Results

### 3.1 Comparison of general data among the three groups

Single factor analysis of variance showed that there were no significant differences in gender, age, systolic blood pressure, diastolic blood pressure, body mass index, glycosylated hemoglobin, uric acid, triglyceride and total cholesterol among the three groups (*p* > 0.05) (Table 1).

**Table 1:** Clinical baseline characteristics of the three groups.

Variables	Non-nephrotic	Microalbuminuria	Macroalbuminuria	X^2^/F	*P*
Age (y)	61.57 ± 7.34	64.67 ± 10.34	66.61 ± 13.26	2.05	0.181
Sex, male/female	18/17	25/24	25/21	3.42	0.134
Total cholesterol (mmol/L)	4.11 ± 1.01	4.39 ± 1.12	4.41 ± 1.35	0.76	0.471
uric acid (μmol/L)	323.25 ± 79.50	320.86 ± 86.89	350.16 ± 108.76	1.20	0.305
Triglyceride (mmol/L)	2.29 ± 1.62	2.44 ± 1.11	2.51 ± 2.48	0.15	0.862
body mass index (kg/m^2^)	25.25 ± 3.35	25.12 ± 1.96	24.80 ± 2.42	0.29	0.747
diastolic pressure (mmHg)	80.63 ± 9.97	79.86 ± 12.75	82.69 ± 9.92	0.69	0.505
Systolic pressure (mmHg)	141.71 ± 14.76	143.45 ± 12.96	143.45 ± 12.96	1.51	0.226
Glycosylated hemoglobin (HbAlc %)	7.74 ± 2.42	8.59 ± 3.09	13.67 ± 31.28	1.27	0.285

### 3.2 Comparison of arterial vascular parameters among the three groups

Table 2 showed the comparison results of retinal artery vascular parameters in the three groups. The statistical results showed that WLR, AWT, and WSCA of the three groups had no statistical significance (*p* > 0.05). The RAOD and RALD of the diabetic non-nephropathy group were 103.69 ± 9.10 μm and 80.34 ± 6.48 μm, which were the largest in the three groups. When the RAOD and RALD of diabetic microalbuminuria group had values of 96.12 ± 11.16 μm and 75.24 ± 10.13 μm, those of diabetic macroalbuminuria group had values of 90.23 ± 9.66 μm and 70.38 ± 13.13 μm. The RAOD and RALD among the three groups were statistically significant (*p* < 0.05).

**Table 2:** Comparison of retinal arteriole parameters among the three groups.

Parameters	RALD	RAOD	WLR	AWT	WSCA
Non-nephrotic	80.34 ± 6.48	103.69 ± 9.10	0.09 ± 0.03	8.81 ± 3.29	3507.11 ± 1203.17
Microalbuminuria	75.24 ± 10.13	96.12 ± 11.16	0.08 ± 0.01	8.07 ± 0.97	3295.85 ± 557.08
Macroalbuminuria	70.38 ± 13.13	90.23 ± 9.66	0.09 ± 0.01	8.32 ± 0.93	3189.40 ± 626.95
F	8.30	15.67	2.53	1.50	1.40
P	<0.05	<0.05	0.084	0.227	0.251

RALD, Rentinal arteriolar lumen diameter; RAOD, Rential arteriolar outer diameter; WLR, wall-to-lumen ratio; AWT, Arteriolar wall thickness; WCSA, Wall cross-sectional area.

### 3.3 Comparison of venous vascular parameters among the three groups

Table 3 presented a comparison of retinal vein parameters across the three groups. The findings indicated that there was no statistically significant difference in terms of WLR, VWT, and WSCA among the groups (*p* > 0.05). Specifically, the RVOD and RVLD measurements for the diabetic non-nephropathy group were 114.17 ± 11.00 μm and 99.69 ± 12.74 μm representing the smallest values among the three groups. In contrast, the RVOD and RVLD measurements for the diabetic microalbuminuria group were 123.73 ± 18.33 μm and 107.05 ± 17.88 μm while those for the diabetic macroalbuminuria group were 130.80 ± 11.70 μm and 115.18 ± 14.25 μm. The differences in RVOD and RVLD among the three groups were found to be statistically significant (*p* < 0.05).

**Table 3:** Comparison of retinal vein parameters among the three groups.

Parameters	RVLD	RVOD	WLR	VWT	VWSCA
Non-nephrotic	99.69 ± 12.74	114.17 ± 11.00	0.06 ± 0.01	6.37 ± 0.78	3468.73 ± 616.71
Microalbuminuria	107.05 ± 17.88	123.73 ± 18.33	0.05 ± 0.01	6.57 ± 0.84	3668.00 ± 673.86
Macroalbuminuria	115.18 ± 14.25	130.80 ± 11.70	0.05 ± 0.01	6.69 ± 0.89	3511.99 ± 772.71
F	8.92	11.56	2.64	1.35	0.99
p	<0.05	<0.05	0.076	0.263	0.373

RVLD, Rentinal venular lumen diameter; RVOD, Rential venular outer diameter; WLR, wall-to-lumen ratio; VWT, Vein wall thickness; WCSA, Wall cross-sectional area.

### 3.4 Correlation between parameters of the retinal arteriole and urine creatinine, microalbumin and UACR in the three groups

Table 4 illustrated the weak association between AWT, WSCA (*R* = 0.190, *p* = 0.037; *R* = 0.210, *p* = 0.021) and Urinecreatinine. Additionally, RAOD (*R* = 0.174, *p* = 0.058), RALD (*R* = 0.125, *p* = 0.174), and WLR (*R* = 0.079, *p* = 0.391) showed no significant correlation with Urinecreatinine. Furthermore, there was no relationship between RAOD, RALD, WLR, AWT, WSCA, Microalbumin, and UACR (*p* > 0.05). The AWT (*R* = 0.020, *p* = 0.827), WLR (*R* = 0.119, *p* = 0.194), WSCA (*R* = 0.089, *p* = 0.334) also did not exhibit a clear correlation with UACR.

**Table 4:** Correlation analysis between parameters of the retinal arteriole and urine creatinine, microalbumin and UACR in the three groups.

Parameters	RAOD	RALD	AWT	WLR	AWSCA
Urinecreatinine					
R	0.174	0.125	0.190	0.079	0.210
P	0.058	0.174	0.037	0.391	0.021
Microalbumin					
R	−0.1390	0.066	0.020	0.099	0.085
P	0.129	0.472	0.827	0.282	0.353
UACR					
R	−0.177	−0.002	0.020	0.119	0.089
P	0.053	0.985	0.827	0.194	0.334

RALD, Rentinal arteriolar lumen diameter; RAOD, Rential arteriolar outer diameter; WLR, wall-to-lumen ratio; AWT, Arteriolar wall thickness; WCSA, Wall cross-sectional area.

### 3.5 Correlation between parameters of the retinal vein and urine creatinine, microalbumin and UACR in the three groups

Table 5 presented the correlation analysis results indicating that RVOD, RVLD, VWT, WLR and WSCA did not show significant correlations with Urinecreatinine. However, RVOD and RVLD exhibited a significant positive correlation with Microalbumin levels (*R* = 0.207, *p* = 0.024; *R* = 0.194, *p* = 0.034). Conversely, VWT, WLR, and WSCA did not show significant correlations with Microalbumin. Additionally, RVOD and RVLD were weakly associated with UACR (*R* = 0.247, *p* = 0.007; *R* = 0.210, *p* = 0.021) while VWT, WLR, and WSCA did not exhibit significant correlations with UACR.

**Table 5:** Correlation analysis between parameters of the retinal vein and urine creatinine, microalbumin and UACR in the three groups.

Parameters	RVOD	RVLD	VWT	WLR	VWSCA
Urinecreatinine					
R	0.021	0.049	−0.134	−0.091	0.117
P	0.824	0.594	0.144	0.322	0.202
Microalbumin					
R	0.207	0.194	0.041	−0.141	0.058
P	0.024	0.034	0.654	0.125	0.532
UACR					
R	0.247	0.210	0.135	−0.113	0.011
P	0.007	0.021	0.142	0.219	0.905

RVLD, Rentinal venular lumen diameter; RVOD, Rential venular outer diameter; WLR, wall-to-lumen ratio; VWT, Vein wall thickness; WCSA, Wall cross-sectional area.

## 4 Discussion

Suboptimal glycemic controlin diabetic patients can lead to chronic vascular complications including diabetic retinopathy and diabetic nephropathy. Chronic high blood sugar causes diabetic microangiopathy damaging the kidney and retina’s small blood vessels. In the kidneys, it alters blood flow, increases filtration pressure and harms glomerular cells and membranes. In the retina, it disrupts the link between capillary cells and the pigment layer, causing vascular leakage and microaneurysms. The emergence of diabetic nephropathy is often associated with changes in the choroid (11–14), retina (15), and optic nerve (16, 17). One study showed that with the increase of UACR, the mean vessel density of the deep retinal capillary plexus decreased significantly in DN (8). In addition, according to a study (9), as the DN lesion degree deepened, not only the deep retinal capillary density decreased, but the shallow retinal capillary density was also significantly reduced. At the same time, Yao and Li (10) also measured the avascular area of the macular fovea and the choroidal vessel density in DN patients and found that the fundus structure of DN changed significantly. They suggested that the enlargement of the avascular area in the fovea might be due to abnormalities in the choroidal vessels and proposed the possibility of macular ischemia While the decrease in choroidal vessel density suggested retinal and choroidal ischemia. High sugar (18), oxidative stress (19), and inflammation factors (20) only aggravate vascular reactions which are thought to be caused by retinal ischemia and hypoxia and the important reason for the change in the structure of the retina. Through checking the retinal blood vessels, fundus and systemic microvascular changes can be more directly and objectively observed in DN patients.

In this study, RAOD and RALD of the diabetic macroalbuminuria group and diabetic microalbuminuria group were significantly lower than those of the diabetic non-nephropathy group. This may be related to damage to the arterial endothelium caused by hyperglycemia (21). Oxidative stress induced by hyperglycemia is believed to cause vascular endothelial damage to retinal arteries in the fundus through the secretion of inflammatory cytokines by infiltrating macrophages (22) leading to vascular blood supply and relaxation dysfunction. At the same time, inflammation promotes fibrosis of the vascular wall and further narrowing of the blood vessels. Microalbuminuria is an early marker of endothelial injury (23). The increase in microalbuminuria not only indicates serious changes in renal pathological structure in patients, but also indicates the continuous increase of endothelial damage to microvessels throughout the body. Feng et al. (24) measured the diameter of retinal microvessels in 690 diabetic patients through an automatic retinal image analysis system and found that narrow retinal arteriolar diameter was positively correlated with the risk of DN in type 2 diabetic patients. This is consistent with the results of this study.

In this study, a large amount of proteinuria was observed in the diabetic microalbuminuria group with RVOD and RVLD being significantly higher than in the diabetic non-nephropathy group. Additionally, the albuminuria group had the highest levels of RVOD and RVLD. This may be related to the increased secretion of vascular endothelial growth factor (VEGF). VEGF (25–27) plays a positive role in promoting vascular endothelial proliferation. Multiple factors (28–32) mediated endothelial damage and VEGF levels increased significantly. These lead to increased permeability of retinal blood vessels, an increase in endovascular blood volume, and an increase in the diameter and flexibility of blood vessels. A high glucose environment leads to decreased deformability of red blood cells in the blood and enhanced aggregation ability (33). Meanwhile, vascular endothelial damage further activates platelets and the physiological mechanism of coagulation (34), thus keeping blood in a hypercoagulable state. This may be another important reason for the increase in retinal vein diameter. Nusinovici et al. (35) measured the vascular diameters of retinal arterioles and venules in 703 white diabetic patients and found that a higher RVOD in white diabetic patients was positively correlated with the risk rate of DN. The findings of this study align closely with those of previous research.

Optical coherence tomography (SD-OCT) combined with semantic segmentation can quickly scan and analyze the parameters of retinal blood vessels. Semantic segmentation is a newly emerging automatic recognition algorithm for identifying, measuring image structure and analyzing vascular parameters. The semantic segmentation method uses deep learning technology to train a large number of retinal blood vessel image data. So that the model can automatically identify and segment the blood vessel regions in the image. During the segmentation process, the algorithm will classify each pixel and label it as a vascular or non-vascular category in order to obtain an accurate blood vessel segmentation result. Based on the segmented vascular regions, the semantic segmentation method can further extract and measure the vascular parameters. By calculating the pixel width of the vessel area, the diameter information of the vessel can be obtained. At the same time, the algorithm can also analyze the direction and tortuosity of blood vessels to evaluate the morphological characteristics of blood vessels. Through the analysis of the overall structure of the network of blood vessels, we can also understand the vascular distribution density and complexity. In practice, semantic segmentation method can be combined with other medical image processing techniques to further improve the accuracy and reliability of retinal vascular parameter measurement.

### 4.1 Advantages and disadvantages

Compared to traditional manual measurement and semi-automatic FWHM image segmentation methods (36, 37), the semantic segmentation method (38) facilitates the automated measurement through artificial intelligence (39–42) mitigating manual errors and enhancing both efficiency and accuracy relative to the semi-automatic FWHM image segmentation method. In practical applications, semantic segmentation method can be integrated with other medical image processing techniques to further augment the accuracy and reliability of retinal vascular parameter measurements (43–45).

The semantic segmentation method can understand the eye lesions more comprehensively. Semantic segmentation method of ophthalmic images allows for a more detailed analysis of lesion areas, which aids in accurate disease evaluation. However, there are some shortcomings. Firstly, the semantic segmentation method is based on artificial intelligence to learn the blood vessel structure parameters and then recognize the blood vessel structure. Manual calculation of blood vessel parameters will inevitably have errors which will directly affect the effectiveness of artificial intelligence learning. Secondly, The semantic segmentation method accurately identifies the vascular structure and measures the vascular parameters by analyzing the pixel differences in the image. Image quality significantly impacts the measurement precision of the segmentation software Thirdly, semantic segmentation method for complex analytical ability of eye disease is limited. Serious eye diseases often have complex pathological mechanisms and intricate texture images. Segmentation based solely on gray level information may not be accurate. This can lead to errors in the segmentation process affecting subsequent analysis and diagnosis. Fourthly, semantic segmentation method has achieved substantial advancements in the domains of image recognition and segmentation. However, it often lacks the sensitivity required to accurately delineate fine boundaries and intricate details in complex images. Conversely, Optical Coherence Tomography Angiography (OCTA) exhibits superior capability in rendering detailed structures and boundaries with greater clarity attributable to its high resolution and significant penetration depth. Furthermore, unlike OCTA which is a non-invasive imaging modality grounded in physical principles and does not necessitate supplementary labeled data, semantic segmentation techniques typically demand extensive pixel-level labeled datasets for effective training. Semantic segmentation method is not only time-consuming and labor-intensive, but also affected by the error of labeling data. Therefore, further improvement of Huiyan software in the later stages requires time to supervise artificial intelligence learning and select a large number of high-definition pictures for artificial intelligence training in order to realize the automatic measurement of vascular structure parameters in a true sense. Considering the aforementioned limitations, there exists substantial potential for future research in the domain of semantic segmentation for the measurement of retinal vessels in patients with diabetic nephropathy. Initially, efforts can be directed toward optimizing the algorithmic model to enhance the accuracy and efficiency of vessel segmentation, thereby facilitating the detection of more nuanced vascular lesions. Secondly, integrating clinical data like renal function and blood glucose is examined to create a comprehensive disease monitoring model. Finally, the study will be converted into clinical tools to aid early diagnosis, disease monitoring, and personalized treatment plans for diabetic nephropath.

In this study, advanced semantic segmentation technology was employed to conduct an in-depth analysis of fundus images from diabetic patients. Retinal blood vessels were automatically segmented and various vascular parameters were successfully extracted. The findings indicate that abnormal changes in these vascular parameters are significantly associated with an elevated risk of diabetic nephropathy. In addition, these studies also show a significant correlation between changes in vascular parameters and progressive progression of diabetic nephropathy. This provides a novel and objective method for the early diagnosis and risk assessment of diabetic nephropathy. This study applies the semantic segmentation method in deep learning to the domain of medical image processing, addressing the limitations inherent in traditional manual measurement techniques and enhancing the accuracy and efficiency of analysis. A notable limitation of this study is the relatively small sample size, which constrains the generalizability and reliability of the findings. This limited sample size may undermine the representativeness of the sample, thereby impeding the extrapolation of the results to the broader population. To address this limitation, future research should consider increasing the sample size to encompass a more diverse cohort of patients with varying backgrounds and degrees of illness severity. Such an approach would enhance the study’s credibility and applicability. Despite the need for further research in model optimization and the exploration of pathophysiological mechanisms, this investigation offers valuable insights and guidance for clinical research and therapeutic practices related to diabetic nephropathy. Furthermore, it underscores the significant potential and promising applications of semantic segmentation method within the medical field.

## Data Availability

The datasets presented in this study can be found in online repositories. The names of the repository/repositories and accession number(s) can be found in the article/Supplementary material.

## Supplementary material

The Supplementary material for this article can be found online at:

https://www.frontiersin.org/articles/10.3389/fmed.2024.1494659/full#supplementary-material
